# Language Barriers in Understanding Healthcare Information: Arabic-Speaking Students’ Comprehension of Diabetic Questionnaires in Arabic and English Languages

**DOI:** 10.7759/cureus.46777

**Published:** 2023-10-10

**Authors:** Zohair J Gazzaz, Mukhtiar Baig, Mohammed Albarakati, Hanady A Alfalig, Tahir Jameel

**Affiliations:** 1 Internal Medicine, Faculty of Medicine, King Abdulaziz University, Jeddah, SAU; 2 Clinical Biochemistry, Faculty of Medicine, King Abdulaziz University, Jeddah, SAU; 3 European Languages, King Abdulaziz University, Jeddah, SAU; 4 Applied Linguistics, King Saud University, Riyadh, SAU

**Keywords:** attitude, knowledge, arabic language, english language, diabetes mellitus

## Abstract

Background

Typically, disease-related information is available in English on the internet, and the bulk of medical research journals are likewise published in English. Therefore, in non-English-speaking countries, many people find it challenging to put that knowledge into practice. This study compared Arabic-speaking students’ performance on a diabetic questionnaire presented in Arabic with their performance on the same questionnaire in English.

Methodology

The cross-sectional study was carried out at the King Abdulaziz University, Jeddah. Identical questionnaires in Arabic and English assessing knowledge, attitude, and practices (KAP) on diabetes mellitus (DM) were filled out by Arabic-speaking students. The English version was distributed to the same students four weeks after the Arabic version. A total of 507 students filled out the Arabic questionnaire, and four weeks later, they filled out the English version.

Results

Students’ scores were significantly higher in the knowledge and attitudes domains (*P *< 0.001), with no significant difference observed in the practice domain on the Arabic language questionnaire compared to English. A gender-wise comparison showed that females had significantly higher knowledge scores in the Arabic and practice domains in the English questionnaires.

According to the regression analysis, students were predicted to have good knowledge scores on the Arabic language questionnaire than on the English version (odds ratio [OR] = 4.537, *P *< 0.001). Similarly, students on the Arabic language questionnaire showed higher scores for positive attitudes (OR = 2.703, *P *< 0.001), and adequate preventative behavior (OR = 1.592, *P *< 0.001) than on the English version. Furthermore, being female is linked to having good knowledge scores (OR = 1.724, *P *< 0.001).

Conclusions

Results indicated that students' good knowledge, positive attitude, and adequate practice scores were associated with the Arabic language questionnaire than the English version. Our participants' KAP scores derived from an English language questionnaire were not up to the mark. There is a need to modify the school curriculum to increase students' English comprehension and command so they can perform better in professional courses.

## Introduction

Usually, disease-related knowledge is available online in English, and most medical research journals are also published in English. Therefore, in non-English-speaking countries, people find it challenging to implement that information in their practice. A study identified literacy skills and the use of non-English (Spanish) language as barriers to online health information seeking among participants [1]. Another study reported that 64.5% of internet users utilize it to find health or medical information. Furthermore, English competence and higher levels of education were favorably connected with seeking internet health information [2].

The English language has undoubtedly attained the status of the dominant language of art, science, medicine, literature, trade, and business worldwide. Achieving proficiency in English is now a necessity in any field of study. Realizing this, the Kingdom of Saudi Arabia (KSA) has long used English as a medium of instruction in professional teaching institutions, including universities [3]. Additionally, the Saudi educational reforms of 2008 further emphasized the importance of English and advocated for increased involvement, leading to the initiation of English instruction in grade four. There is also some discussion of a plan to commence teaching English in grade one next year.

Most students enrolled in various faculties at King Abdulaziz University (KAU) come from the high school education system in which teaching has taken place almost entirely in Arabic, except English is taught as a subject. Teaching English in these schools is still at a basic level taught by Arab and Saudi native speakers. This, among other reasons, has made students generally weak and unable to comprehend professional subjects' technicalities [4]. Thus, for newly inducted medical students, English teaching in the preparatory year is a standardized course covering all aspects of English acquisition, including terminology, speaking, writing, and comprehension [5]. During the preparatory year, students progress through the basic level to higher levels by institutional assessments based on their achievement in all skills (listening, speaking, reading, and writing) [6]. Therefore, students who have attended English schools in the KSA or other countries usually score higher on the placement test and are placed in higher levels at the start of the preparatory year. This gives them the advantage of not having to spend time learning the basic aspects of the language [7].

In the Saudi Arabian teaching system, Arabic is the primary medium of expression, and certain teachers voice their concerns regarding learners’ motivation levels. Low motivation makes it difficult to achieve the required proficiency level in the short period of the preparatory year; other essential science subjects need to be taught in parallel during that time. Many factors are operative when beginning to learn a second language. Arabic is a very strong language with deep-rooted effects on society and is the official language in almost all governmental offices. Moreover, the education process is carried out primarily in Arabic and allows little space for other languages [8]. Previous research has probed the issues linked to this problem; some have indicated a lack of interest in learners. In contrast, others have pointed to flaws in teaching methodology lack of proper training of teachers, irrelevant teaching qualifications, and lack of experience as bilingual teachers [3,9,10].

A study regarding learning resources indicated that medical students often avoid textbooks because of difficulties in understanding English. They find difficulty responding to short essay questions and presenting cases in class discussions difficult [11]. Several studies have reported Saudi students' challenges and difficulties when studying in international settings. They concluded that language becomes a major barrier for these students to participate in class discussions, group presentations, and responding to essay-type questions [12,13].

The preparatory year provides a good opportunity for newly inducted students at KAU to improve their English as a Foreign Language (EFL) learning. In this context, this study was designed to identify gaps in Arabic-speaking students’ grasp of the English language by comparing their scores on an Arabic language questionnaire to their scores on the same questionnaire presented in the English language, assessing their KAP on diabetes mellitus (DM). Assessing the Arabic-speaking students’ understanding of the English questionnaire and uncovering specific shortcomings can point to ways to improve the English-teaching methods.

## Materials and methods

Study cohorts

We conducted this study on the Rabigh Campus, KAU. Rabigh Campus is a comparatively new branch situated 160 km from Jeddah. Ethical approval was obtained from the Faculty of Medicine, Rabigh Campus, Research Ethics Committee (Ethical approval no. RMC-01-40-H). All participants were informed about the survey's objective, and their written consent was obtained before the start of the study. This study included 507 freshly enrolled students completing the preparation year training at the Rabigh Campus. Like many Saudi universities, KAU offers a one-year preparatory course to all students admitted to the university's various faculties. In preparatory year courses, science-track students are taught *English, physics, chemistry, and biology*. After the year, students are accepted into their majors based on their performance in the prep year.

Study tool

This study used an already-validated diabetic questionnaire that has been used in several studies. The questionnaire was originally in English, and two bilingual experts translated and back-translated it (English/Arabic/English). It was then updated according to their recommendations. We only did content validation of the Arabic translation. The researchers performed a preliminary survey with 35 respondents to verify the clearness of the survey form (Cronbach's alpha was 0.75). The questionnaire contained sections comprising demographic details and questions regarding KAP on DM.

Questionnaires in Arabic and English included "43, 11, and 10 questions related to knowledge, attitudes, and practices, respectively. The scoring of the knowledge items was as follows: up to 50% = poor, and above 50% = good. The attitude scoring was as follows: correct answer = 1 point, incorrect (no) = minus score, unsure = 0 points. A plus score was considered positive, while a 0 or a minus score was considered negative. The practice questions were coded as correct (yes) answer = 1 point, incorrect (no) = 0 points, unsure = 0 points; above 50% was considered adequate" [14]. The Arabic version was given to female and male students in the initial phase. Students were asked to write their names and university ID numbers on the questionnaire. The English version was distributed to the same students four weeks later for their responses. The responses of the questionnaire's Arabic and English versions were segregated and then analyzed regarding students' KAP scores. 

Statistical analysis

Data were analyzed using IBM SPSS Statistics for Windows, Version 26.0 (IBM Corp., Armonk, NY) software. The descriptive analysis was performed, and data were represented as frequencies and percentages. The KAP scores were calculated and presented as mean and standard deviation. The binary logistic regression analysis was utilized to compute associations of the questionnaire language with KAP scores. All *P*-values < 0.05 were considered significant.

## Results

A total of 507 students (274 females, 54%, and 233 males, 46%) filled out the Arabic questionnaire and, four weeks later, filled out the English version. All participants were preparatory year students, divided into four levels according to their English language proficiency according to the preparatory year administration. Their general characteristics are given in Table 1. 

**Table 1:** General characteristics of the participants who filled out Arabic and English language questionnaires. The data have been represented as frequency (*N*) and percentage (%). ^*^All participants filled out the questionnaire twice; therefore, values are the same in both columns. ^^^Students were divided into four categories in preparatory years according to their English levels.

Variables		Arabic questionnaire^*^ *N* = 507 (%)	English questionnaire *N* = 507 (%)
Gender	Female	274 (54)	274 (54)
	Male	233 (46)	233 (46)
English level^^^	1	56 (11)	56 (11)
	2	325 (64.1)	325 (64.1)
	3	86 (17)	86 (17)
	4	40 (7.9)	40 (7.9)

The students scored significantly better in knowledge (*P* < 0.001) and attitudes (*P* < 0.001), while no significant difference was observed in practice scores between the Arabic and English language questionnaires (Table 2).

**Table 2:** Comparison of Arabic and English language questionnaires according to participants’ knowledge, attitudes, and practice scores. The data have been represented as mean ± standard deviation (SD). ^*^P-value <0.05 was considered significant.

Variables	Arabic, *N* = 507	English, *N* = 507	*P*-value
Knowledge score	27.01 ± 8.06	20.47 ± 8.90	<0.001^*^
Attitude score	5.67 ± 4.76	3.42 ± 4.70	<0.001^*^
Practice score	5.86 ± 2.85	5.63 ± 2.77	0.204

We compared the KAP scores according to the student's English levels and found that level 3 students had significantly better knowledge than those in level 2 (*P* = 0.006). In contrast, no difference was found among other levels (Table 3).

**Table 3:** Comparison of students’ knowledge, attitudes, and practice scores according to their English level. Data have been represented as mean ± standard deviation (SD). ^^^The *F*-test for analysis of variance (ANOVA). ^^^^Multiple comparisons between each two categories by post hoc analysis (Bonferroni test). ^*^P-value <0.05 was considered significant. NS, not significant

Variables	English level	N	Mean ± SD	*P*-value
DM total score Knowledge	1	112	23.68 ± 10.24	*F*^^^ = 5.57 (P^*^ = 0.001), level 2^^^^ versus level 3 (P^*^= 0.006)
	2	650	23.03 ± 9.30	
	3	172	26.13 ± 7.05	
	4	80	24.51 ± 8.81	
Attitude	1	112	4.46 ± 6.05	*F*^ = 0.981 (*P *= 0.401) NS
	2	650	4.42 ± 4.80	
	3	172	4.69 ± 4.56	
	4	80	5.37 ± 4.10	
Practices	1	112	5.44 ± 3.33	*F*^^^ = 2.04 (*P *= 0.106) NS
	2	650	5.68 ± 2.88	
	3	172	6.20 ± 2.18	
	4	80	5.72 ± 2.63	

A gender-wise comparison was carried out on participants' KAP scores on the Arabic and English questionnaires. It showed that females had significantly higher knowledge scores in the Arabic questionnaire and practices in the English questionnaire (Table 4).

**Table 4:** Gender-wise comparison of Arabic and English language questionnaires according to participants’ knowledge, attitudes, and practice scores. The data have been represented as mean ± standard deviation (SD). ^*^P-value <0.05 was considered significant.

Variables		Knowledge score	Attitude score	Practice score
Arabic questionnaire	Female (*N* = 274)	28.13 ± 7.82	6.18 ± 4.79	5.86 ± 2.92
	Male (*N* = 233)	25.71 ± 8.16	5.06 ± 4.66	5.86 ± 2.78
	*P*-value	0.001^*^	0.008^*^	0.996
English questionnaire	Female (*N* = 274)	21.17 ± 9.82	5.78 ± 2.92	4.11 ± 4.94
	Male (*N* = 233)	19.65 ± 7.62	5.46 ± 2.58	2.61 ± 4.28
	*P*-value	0.05	0.201	<0.001^*^

We compared the KAP scores according to students' English levels and found that level 3 students had significantly better knowledge than those in level 2 (*P* = 0.006). In contrast, no difference was found among students at other levels (Table 3).

The regression analysis showed that students were expected to have good knowledge scores on the Arabic language questionnaire than on the English version (odds ratio [OR] = 4.537, *P *< 0.001). Similarly, students tend to have positive attitudes scores (OR = 2.703, *P *< 0.001) and adequate preventive practice scores (OR = 1.592, *P *< 0.001) on the Arabic language questionnaire than on the English version. Additionally, being female is associated with good knowledge scores (OR = 1.724, *P *< 0.001) but does not significantly influence attitudes and practices in this context. There was no significant association between English levels and scores (Table 5).

**Table 5:** Association of questionnaire language with knowledge, attitudes, and practice scores (binary logistic regression analysis). ^*^P-value <0.05 was considered significant. Knowledge: up to 50%, poor score; >50%, good score. Attitude: plus score, positive; minus score, negative. Practice: up to 50% score, inadequate; >50%, adequate.

Variables	Knowledge			Attitudes			Practice		
	B	*P*-value	OR	B	*P*-value	OR	B	*P*-value	OR
Questionnaire language									
Arabic	1.470	<0.001^*^	4.348	0.994	<0.001^*^	2.703	0.465	<0.001^*^	1.592
English (by level)									
1	–0.034	0.918	0.967	–0.962	0.053	0.382	–0.150	0.619	0.861
2	–0.348	0.191	0.706	–0.830	0.061	0.436	–0.190	0.441	0.827
3	0.421	0.177	1.523	–0.574	0.240	0.563	0.471	0.106	1.602
Female	0.545	<0.001^*^	1.724	0.315	0.081	1.371	0.137	0.304	1.146

## Discussion

Learning a second language is a complex process involving many factors determining the success or failure of the whole process. As a bilateral process, students and teachers are equally involved. Students' interest and motivation and the teacher's commitment, confidence, teaching methodology, and experience are very important [13]. If the teacher guides students through the technical details and provides encouragement to use the second language in conversation, it can produce outstanding results [15]. Students start learning a second language more rapidly when there is no other option but to use the language being learned to make others understand their point of view [16]. This was not the situation with our students, who continued using Arabic (their native language) in daily conversation. We adopted an interesting modus operandi to compare our students' KAP regarding DM using a questionnaire in the Arabic language and then assigning it in English after a significant gap when most students would have forgotten their previous responses to the Arabic questionnaire. Thus, a comparison of their responses reflects their grasp of the English language.

Arabic being our students' mother tongue, the KAP scores were significantly higher on the Arabic version than on the English version. Two recent studies involving native-speaking Arabs revealed that understanding and KAP scores were considerably better on the Arabic version than the English questionnaire. Students experienced difficulties understanding the technicalities of materials published in English [17]. Two studies have pointed out that individuals who have a disease or have a near relative with it have better knowledge and practices against that disease than healthy individuals [18,19]. Our female preparatory year participants had significantly better KAP scores statistically as compared to male students. Binary logistic regression analysis also revealed that the Arabic version of the questionnaire yielded good KAP scores.

Association of questionnaire language with KAP scores showed that compared to English, Arabic was associated 4.348, 2.703, and 1.592 times with good knowledge, positive attitude, and adequate practice scores, respectively. According to a study, taking the exam in a language in which the participants were not fluent appears to have had a detrimental impact on the test's outcome [20]. 

Zakarneh et al. pointed out many challenges in teaching EFL to mixed-ability classes of Arab students [21]. The majority of the students come from schools with a pure Arabic setup (slow learners) and some from English medium schools (fast learners). Slow learners are often not very interested in learning EFL. With additional time devoted to slow learners, fast learners may get bored and lose interest [21]. Our study participants were divided into four levels according to English proficiency. Participants who studied in schools in which the mode of instruction was their native language were placed in level 1/2. Students who attended English medium schools were fluent in English and were placed in level 3/4. Our results showed better KAP scores of level 3 English students (*P* < 0.001). Wallin and Cheevakumjorn pointed out important findings. First, if students start learning English early, they excel much better in professional studies because they more easily grasp instruction material written mainly in English [22]. They are better in class discussions and presentations. Second, if students are compelled to converse only in the second language, learning will be faster and better.

Massri discussed the factors affecting EFL in KSA, both student- and teacher-related factors. Student-related factors include social pressure, family expectations, propositions, and their goals to move abroad for higher studies [23]. Another recent study investigated the reasons for indifference to learning EFL. It noted a few important observations, such as teachers not being motivators or having qualifications irrelevant to their job [24]. Experienced teachers do not often adopt newer modes of teaching. Most teachers who are native English speakers are either not recruited or the facilities offered do not attract them. The result is that English teachers from neighboring countries find the job lucrative to fill the vacancies [24,25]. Division of the participants according to their English proficiency proved useful in our study as the students in, for example, level 3 were 71% more likely to have good knowledge scores compared to those from lower levels. The analysis found Similar trends for attitudes and practices, though no statistical significance was observed.

Arabic is a very rich language, and Saudi people take pride in their mother tongue. Because it is the official language in the Kingdom, it is not easy to find English-speaking individuals in many official dealings. With advancements in all areas of life, people are now becoming interested in learning EFL, especially the young [26]. Alsahafi and Shin have mentioned major challenges in Saudi international students' academic and cultural adjustment in Australian universities [27]. Lack of English language proficiency was found to be the foremost factor leading to difficulties in adjusting to an English-speaking society, class discussions, and completing written assignments. When students do not meet the expectations of their teachers and fellow students, it can cause anxiety, stress, and depressive effects, often requiring psychiatric consultation [28]. Our study participants are at the initial phase of their professional training; they will improve their English language proficiency with proper counseling and motivation. Command of the English language will help them participate confidently in their professional activities and attain successful careers in the future. Smartphone applications can be very effectively utilized to familiarize students with the English language. Previous studies have revealed the addictive effect of mobile apps on students, leading to definite improvement in their English language learning and understanding [29,30].

Based on these study results, it is suggested that information regarding DM and other important problems, including COVID-19, should be provided in the national language so that people can comprehend better and act accordingly. There is also the need for cooperation between high school teachers and KAU English administrators to prepare students for EFL by conducting workshops in schools or universities to raise students' awareness of the importance of the preparatory year and of learning English. A study skills course can also be added to the prep year program to familiarize students with the essential skills required to excel at universities, including class discussions, note-taking, and presentation skills (all offered in English). Moreover, learning English is one of the goals of the Saudi 2030 vision.

This study has a few limitations, including its small sample size and the fact that all students were in only the preparatory year. Our results cannot be generalized due to having been conducted on one campus of KAU; it is likely that students of the Jeddah campus and other universities in the Kingdom may better understand the English language.

## Conclusions

Our participants scored differently on both questionnaires. Our participants' KAP scores deriving from an English language questionnaire were not up to the mark. Results indicated that students' good knowledge, positive attitude, and adequate practice scores were associated with the Arabic language questionnaire than the English version.

There is a need to modify the curriculum at the school level so that students' comprehension and command of the English language improve, and they can qualify better in understanding and performing in their professional studies. This would be very helpful for those who plan to study abroad for advanced and postgraduate education.

## References

[1] Millar RJ, Sahoo S, Yamashita T, Cummins PA (2020). Literacy skills, language use, and online health information seeking among Hispanic adults in the United States. Patient Educ Couns.

[2] Nguyen A, Mosadeghi S, Almario CV (2017). Persistent digital divide in access to and use of the Internet as a resource for health information: Results from a California population-based study. Int J Med Inform.

[3] Shah SR, Hussain MA, Nasseef OA (2013). Factors impacting EFL teaching: an exploratory study in the Saudi Arabian context. Arab World Engl J.

[4] Louber I, Troudi S (2019). “Most of the Teaching is in Arabic Anyway”, English as a medium of instruction in Saudi Arabia, between de facto and official language policy. Int J Bias Identity Diversities Educ.

[5] Al-Ahdal AH, Alfallaj F, Al-Awaied S (2014). A comparative study of proficiency in speaking and writing among EFL learners in Saudi Arabia. Am Int J Contemp Res.

[6] Mubina R (2015). Best Practices in English Language Testing at the University Preparatory Year Programs. Teaching and Learning in Saudi Arabia: Perspectives From Higher Education.

[7] McMullen MG (2014). The value and attributes of an effective preparatory English program: perceptions of Saudi university students. Engl Lang Teach.

[8] Fareh S (2010). Challenges of teaching English in the Arab world: why can’t EFL programs deliver as expected?. Procedia Soc Behav Sci.

[9] Alnujaidi S (2019). The difference between EFL students' preferred learning styles and EFL teachers' preferred teaching styles in Saudi Arabia. Engl Lang Teach.

[10] Kassem HM (2019). The impact of student-centered instruction on EFL learners' affect and achievement. English Language Teaching.

[11] Jameel T, Baig M, Gazzaz ZJ, Tashkandi JM, Al Alhareth NS, Khan SA, Butt NS (2019). Approaches towards professional studies and spare-time activities among preclinical and clinical year medical students. Cureus.

[12] Alharbi MA (2019). Saudi Arabia EFL university students’ voice on challenges and solution in learning academic writing. Indones J Appl Linguist.

[13] Namaziandost E, Neisi L, Nasri M (2019). Enhancing oral proficiency through cooperative learning among intermediate EFL learners: English learning motivation in focus. Cogent Educ.

[14] Gazzaz ZJ (2020). Knowledge, attitudes, and practices regarding diabetes mellitus among university students in Jeddah, Saudi Arabia. Diabetes Metab Syndr Obes.

[15] Portolés L, Martí O (2020). Teachers’ beliefs about multilingual pedagogies and the role of initial training. Int J Multiling.

[16] Howard M (2019). An Introduction to the Volume. Second Language Acquisition and Interculturality During Study Abroad: Issues and Perspectives.

[17] Suleiman K, Al Kalaldeh M, AbuSharour L (2019). Validation study of the Arabic version of the Brief Fatigue Inventory (BFI-A). East Mediterr Health J.

[18] Sharma S, Jha J, Varshney A (2020). Awareness of various aspects of diabetes among people visiting tertiary eye care institute in north India. Clin Epidemiology Glob Health.

[19] Shim JS, Heo JE, Kim HC (2020). Factors associated with dietary adherence to the guidelines for prevention and treatment of hypertension among Korean adults with and without hypertension. Clin Hypertens.

[20] Jacobson HE, Hund L, Soto Mas F (2016). Predictors of English Health Literacy among US Hispanic Immigrants: the importance of language, bilingualism and sociolinguistic environment. Lit Numer Stud.

[21] Zakarneh B, Al-Ramahi N, Mahmoud M (2020). Challenges of teaching English language classes of slow and fast learners in the United Arab Emirates universities. Int J High Educ.

[22] Wallin J, Cheevakumjorn B (2020). Learning English as a second language: earlier is better. J Eng Educ Soc.

[23] Massri R (2019). The perceptions and beliefs of Saudi preparatory year program learners towards learning English: a case study. Arab World Engl J.

[24] Alqahtani M (2019). The effect of native English-speaking teachers on the Language acquisition of EFL learners. Int J Innov Res Educ Sci.

[25] Shamim F (2019). Proficiency and professionalism: Arab teachers' perceptions of professional identity in Saudi Arabia. Kashmir J Lang Res.

[26] Tatar S (2019). Employment of English language teachers in an EFL context: perspectives from school administrators. Profile: Issues Teach Prof Dev.

[27] Alsahafi N, Shin SC (2019). Factors affecting the academic and cultural adjustment of Saudi international students in Australian universities. J Int Stud.

[28] Gazzaz ZJ, Baig M, Al Alhendi BS, Al Suliman MM, Al Alhendi AS, Al-Grad MS, Qurayshah MA (2018). Perceived stress, reasons for and sources of stress among medical students at Rabigh Medical College, King Abdulaziz University, Jeddah, Saudi Arabia. BMC Med Educ.

[29] Alhazmi AA, Alzahrani SH, Baig M, Salawati EM, Alkatheri A (2018). Prevalence and factors associated with smartphone addiction among medical students at King Abdulaziz University, Jeddah. Pak J Med Sci.

[30] Sayedalamin Z, Alshuaibi A, Almutairi O, Baghaffar M, Jameel T, Baig M (2016). Utilization of smart phones related medical applications among medical students at King Abdulaziz University, Jeddah: a cross-sectional study. J Infect Public Health.

